# Competitive Foods’ Nutritional Quality and Compliance with Smart Snacks Standards: An Analysis of a National Sample of U.S. Middle and High Schools

**DOI:** 10.3390/nu16020275

**Published:** 2024-01-17

**Authors:** Juliana F. W. Cohen, Ashley Kesack, Tara P. Daly, Sara A. Elnakib, Erin Hager, Samuel Hahn, Daniel Hamlin, Alla Hill, Annie Lehmann, Peter Lurie, Meghan Maroney, Jaydn Means, Megan P. Mueller, Deborah A. Olarte, Michele Polacsek, Marlene B. Schwartz, Kendrin R. Sonneville, Lori A. Spruance, Andrea R. Woodward, Leah E. Chapman

**Affiliations:** 1 Center for Health Inclusion, Research and Practice (CHIRP), Department of Nutrition and Public Health, Merrimack College, 315 Turnpike Street, North Andover, MA 01845, USA; kesacka@merrimack.edu (A.K.); dalyt@merrimack.edu (T.P.D.); olarted@merrimack.edu (D.A.O.); chapmanle@merrimack.edu (L.E.C.); 2 Department of Nutrition, Harvard T.H. Chan School of Public Health, 677 Huntington Ave, Boston, MA 02115, USA; 3 Department of Family and Community Health Sciences, Rutgers University, New Brunswick, NJ 08901, USA; elnakisa@njaes.rutgers.edu; 4 Department of Population, Family and Reproductive Health, Johns Hopkins Bloomberg School of Public Health, 615 N. Wolfe Street, Baltimore, MD 21205, USA; ehager1@jhmi.edu; 5 Center for Science in the Public Interest, Washington, DC 20005, USAahill@cspinet.org (A.H.); plurie@cspinet.org (P.L.); mmaroney@cspinet.org (M.M.); 6 Department of Educational Leadership and Policy Studies, University of Oklahoma, Tulsa, OK 74135, USA; daniel_hamlin@ou.edu (D.H.); jaydnmeans@ou.edu (J.M.); 7 School of Allied Health, University of Alaska Anchorage, Anchorage, AK 99508, USA; amlehmann@alaska.edu; 8 Department of Food Science and Human Nutrition, Colorado State University, 1571 Campus Dr, Fort Collins, CO 80523, USA; megan.mueller@colostate.edu; 9 Center for Excellence in Public Health, University of New England, 716 Stevens Ave, Portland, ME 04103, USA; mpolacsek@une.edu; 10 Rudd Center for Food Policy and Health, Department of Human Development and Family Sciences, University of Connecticut, 1 Constitution Plaza, Suite 600, Hartford, CT 06103, USA; marlene.schwartz@uconn.edu; 11 Department of Nutritional Sciences, University of Michigan School of Public Health, Ann Arbor, MI 48109, USA; kendrins@umich.edu; 12 Department of Public Health, Brigham Young University, Provo, UT 84602, USA; lori.spruance@byu.edu; 13 Sociology Department, Berea College, Berea, KY 40404, USA; woodwarda@berea.edu

**Keywords:** competitive foods, Smart Snacks, school policy, universal free school meals, nutrition

## Abstract

Snacks and beverages are often sold in addition to meals in U.S. schools (“competitive foods”), but their current nutritional quality and compliance with national Smart Snacks standards are unknown. This study assessed competitive foods in a national sample of 90 middle and high schools. Differences in compliance by school characteristics were measured using mixed methods analysis of variance. Overall, 80% of the schools in the sample sold competitive foods; but they were less commonly available in schools with universal free school meal (UFSM) policies. A total of 840 unique products were documented and, on average, 75% were compliant with Smart Snacks standards. A total of 56% aligned with recommended added sugar limits (<10% of calories); and 340 unique products (40%) aligned with both sugar and Smart Snacks standards. Approximately one-fifth of competitive foods contained synthetic dyes, and 31% of beverages contained artificial sweeteners. Smart Snacks standards compliance was greater when competitive foods were overseen by food service departments, in comparison with others (e.g., principals, student organizations, or outside vendors [77% vs. 59% compliance; *p* = 0.003]). Therefore, district wellness policies should consider requiring food service departments to oversee competitive foods. Federal and state policies should limit added sugars, artificial sweeteners, and synthetic dyes. This appears to be highly feasible, given the substantial number of products that meet these criteria. UFSM policies should also be considered to support healthier school meal environments more broadly.

## 1. Introduction

Many schools in the United States (U.S.) sell snacks and beverages (“competitive foods”) [1]. These often “compete” with school meals offered in the School Breakfast Program and the National School Lunch Program. Potentially, this undermines nutritional advances resulting from improved nutrition standards ushered in by the Healthy, Hunger-Free Kids Act (HHFKA) of 2010 and the ensuing U.S. Department of Agriculture (USDA) regulations [1]. Prior to the implementation of federal standards for competitive foods, 82% to 97% of middle- and high-school students had access to snacks and beverages in vending machines, à la carte on cafeteria lines, and in school stores [2]. These competitive foods tended to be of poor nutritional quality and high in calories, saturated fat, and total sugar [3,4,5,6]. Additionally, competitive food consumption was associated with reduced consumption of healthier foods—including fruits and vegetables—both during and outside the school day [6,7]. 

In 2014, the USDA implemented Smart Snacks in School (“Smart Snacks”) as national mandatory standards to improve the nutritional quality of competitive foods [8]. These standards emphasized whole grains, fruits, vegetables, and low-fat dairy; limited calories, sodium, saturated fats, and total sugar (with the exception of naturally occurring sugar such as that in fruit, as well as dried fruit with added sugars); and eliminated trans fats [8]. Preliminary research conducted immediately following implementation of the Smart Snacks standards suggested these regulations for competitive foods improved the overall nutritional quality of the snacks and beverages sold in schools [9]. 

After the Smart Snacks standards were implemented, several issues relevant to the nutrition standards for competitive foods emerged. First, there is growing evidence regarding adverse health outcomes associated with added sugar consumption, and the two most recent Dietary Guidelines for Americans (DGAs) recommend that less than 10% of calories come from added sugars [10,11,12]. While the HHFKA set limits on total sugars for Smart Snacks—requiring that total sugar should not account for more than 35% of the product’s weight—this standard requires considerable calculations, and this makes it difficult to implement. Furthermore, it does not expressly limit added sugars. Since 1 January 2021, all nutrition facts labels are required to list added sugars [13] and, therefore, updating the standards for competitive foods to address added sugars specifically is now feasible. 

Second, Smart Snacks standards allow beverages with non-sugar sweeteners (including artificial and natural sweeteners) to be sold in high schools, but they prohibit selling these same beverages in elementary and middle schools. However, the safety of non-sugar sweeteners—sometimes called low-calorie sweeteners (LCS), non-nutritive sweeteners (NNS), or high-intensity sweeteners—has been the subject of substantial debate. This is particularly the case for artificial non-sugar sweeteners. The U.S. Food and Drug Administration (FDA) has concluded that the artificial sweeteners currently on the market are safe, and it permits their use in food [14]. In contrast, in 2018, an American Heart Association Science Advisory concluded, “it is prudent to advise against prolonged consumption of LCS beverages by children” [15]. In 2019, the American Academy of Pediatrics concluded, “the long-term safety of NNS in childhood has not been assessed in humans” [16]. There is particularly compelling evidence that aspartame is a carcinogen, with the World Health Organization recently classifying aspartame as “possibly carcinogenic to humans” [17]. Further, emerging evidence suggests that non-sugar sweeteners may be ineffective for weight control in children; this includes a recent World Health Organization (WHO) report that stated “that non-sugar sweeteners not be used as a means of achieving weight control” based on “no evidence of long-term benefit on measures of body fatness in adults or children” [18]. Despite this, there is evidence that manufacturers are increasingly including non-sugar sweeteners in products for both children and adults [16]. 

Lastly, synthetic (i.e., artificial) food dyes and other additives are pervasive among children’s products in the U.S.’s food supply, but they are not currently regulated by the Smart Snacks standards [19]. There is emerging evidence that some synthetic dyes may be associated with neurobehavioral problems in some children [20]. In Europe, products that contain certain synthetic dyes are required to have a warning label stating that these synthetic dyes “may have an adverse effect on activity and attention in children”, and the state of California recently banned several additives, including Red No. 3, due to the potentially increased risk of cancer [21]. Additionally, there is growing evidence that certain additives, including titanium dioxide and tert-butylhydroquinone (TBHQ), may be associated with increased cancer risk; furthermore, based on the strength of the evidence, titanium dioxide has been banned from the European Union [22,23]. Therefore, there is a need to assess the current landscape of competitive foods in the U.S., including the quantity of added sugars and the presence of artificial sweeteners, food dyes, and other additives. 

In the decade since the Smart Snacks regulations were released, manufacturers have developed many Smart Snacks-compliant products [24]. In recent years, however, the COVID-19 pandemic may have influenced the availability and compliance of competitive foods due to staffing concerns and supply chain challenges [25]. Therefore, there is a need for a comprehensive assessment of compliance with the Smart Snacks standards. Drawing from a national sample of middle and high schools, this study had two objectives: (1) to assess the extent of compliance with Smart Snacks standards; and (2) to investigate the amount of added sugars, and the presence of non-sugar sweeteners (including artificial and natural sweeteners), food dyes, and selected other additives in competitive foods. 

## 2. Materials and Methods

### 2.1. Recruitment of Schools

Two states from each of the seven USDA Food and Nutrition Service (FNS) administrative regions (Supplementary Figure S1) were randomly selected to participate in the study. New Jersey is a state with an expanded eligibility threshold for free school meals (200% of the federal poverty line). It was added to the study sample to ensure representation among differing state-level school meal policies. This resulted in a sample of 15 states. A comprehensive list of school districts within each of the participating states was obtained through the National Center for Education Statistics (NCES) [26]. To support the feasibility of in-person data collection within larger states, school districts had to be within a 90 min drive for research assistants (RAs); these were hired through local universities within target states. 

School districts within the sampled states were selected using stratified random sampling by urbanicity to ensure one rural, one suburban, and one urban school district was included within each state. The goal was to recruit 45 school districts in total. Urbanicity was determined using Rural-Urban Commuting Area (RUCA) codes acquired from the USDA Economic Research Service [27]. The RUCA codes were collapsed into three categories: 1 = urban; 2–3 = suburban; and 4–10 = rural. Study team members reached out to the food service directors and principals in the selected school districts. They typically sent two to three rounds of recruitment emails per potential district and then followed up via telephone. If a school district declined to participate, another district was randomly selected from within the same stratum for urbanicity within the state. A total of 155 school districts were contacted, to yield the 45 districts (the participation rate was 29%). Overall, districts that declined to participate were similar to those that agreed to participate with respect to the percentage of students eligible for free or reduced-price meals (FRPM) as well as their likelihood of having free school meal policies. 

Among the participating districts, one middle and one high school were then randomly selected to participate in the study, to make a total sample of 90 schools. To limit data collection burden in New Jersey, the three school districts were recruited from elementary school-aged children who were enrolled in an ongoing study that was unrelated to competitive foods. 

Food service directors from all the participating districts completed a brief online survey, with separate questions for middle and high schools. The survey included questions on the percentage of students who were eligible for FRPM, whether the school provided free school meals to all students through policies such as the Community Eligibility Provision (CEP) or state-level UFSM policies, whether there were open campuses (i.e., students could leave during lunch hours), and who oversaw the operation of competitive foods at the differing locations (i.e., food service, outside vendors, principal, student organizations, or others). This study was deemed exempt by Merrimack College’s Institutional Review Board (protocol # IRB-FY22-23-41).

### 2.2. Competitive Foods

To document the availability of competitive foods through direct observation, site visits were conducted in the spring of 2023 and employed methods consistent with those established in previous research [9,28]. The research team worked with each high school and middle school’s cafeteria manager or staff, to ensure that all locations with competitive foods in a school were identified and visited during the site visits. The number of vending machines and their locations (e.g., inside cafeteria, outside cafeteria, in hallways, etc.) were also recorded. Each vending machine, à la carte line, and/or school store was counted as a separate location. During site visits, trained research assistants took digital photographs of all the snacks and beverages available in vending machines, on food service à la carte lines, and in school stores. The competitive foods were photographed so that the product name, brand, size, and location within the school could be clearly viewed. Individually wrapped foods, fruits and vegetables sold outside of the school meal program (such as in vending machines) and baked goods prepared on site by the food service department and sold at cash registers were included in the study. The total number of competitive foods offered was calculated for each location (e.g., if the same product was offered in two vending machines, and in the à la carte line in the cafeteria within a given school, it was counted as being offered “3 times”). Competitive foods were also categorized by product type—including beverages, salty snacks, sweet snacks, ice cream or frozen treats, yogurt or cheese, and fruits or vegetables—using previously developed methods [9,28].

Information on the timing of access to the competitive foods was recorded and coded as being available during school hours, only before school, and/or only after school (e.g., some vending machines had timed locks that only enabled students to purchase these snacks or beverages outside of school hours). Competitive foods that were not available to students during school hours were excluded. Similarly, vending machines in teachers’ lounges were excluded if students did not have access to them. Foods and beverages that were provided to students as part of the school breakfast or lunch program, and which the school did not list as a competitive food or for which the school did not provide an individual price as a competitive food, were also excluded from the study. 

Nutrition information for each competitive food was obtained from the product’s nutrition facts label, when visible from photographs, or the manufacturer’s website when nutrition labels were not visible (e.g., in vending machines). When manufacturers’ websites did not have nutrition information, the nutrition information was obtained and verified from at least three third-party retailer websites that sold the products (e.g., Amazon, Target, Walmart) by cross-referencing across retailers’ websites product attributes such as package size, serving size, and ingredient lists. Nutrition information from the original packaging of foods baked on site (e.g., the nutrition facts label from the original cookie dough box) was provided by the cafeteria managers. 

Manufacturers are also known to create “copycat” versions of products to comply with the Smart Snacks standards; one version is formulated to meet the standards and is only sold in schools, and the other version may not meet the standards and is sold in retail locations outside of schools [29]. These products are often nearly identical in packaging and name, but they may have different ingredients and nutrition profiles. The photographs of competitive foods were, therefore, examined to ensure that nutrition information was recorded for the correct product. 

The nutrients and the associated Smart Snacks standards for foods examined were: total calories (≤200 kcal); percentage of calories from total fats (≤35% of calories); percentage of calories from saturated fats (<10% of calories); trans fat (0 g); sodium (≤200 mg); and total sugars (≤35% by product weight). Nutrition label ingredient lists were also examined for whole grains, where applicable. For competitive beverages, USDA defines compliant drinks as water (plain, with or without carbonation); juice or milk (≤12 oz); and low-calorie or no-calorie drinks (at the high-school level only). Fiber, added sugars, and percentage of calories from added sugars for each product were also examined, although these are not part of current Smart Snacks standards. Approximately 6% of products were missing information on added sugars, even after examining multiple product websites. The nutrition facts per serving and the number of servings per package were used to calculate the total nutrients per package. 

All products were examined for full compliance with the Smart Snacks standards, as well as by each Smarts Snacks requirement for nutrients, serving sizes for beverages, etc. Products were also examined to see if they were in alignment with DGA recommendations, which say that less than 10% of calories should come from added sugars. This was calculated based on the calorie content of individual products (whereas the DGA recommendations are based on total daily calories). Nutrition labels were also examined for the presence of non-sugar sweeteners, including artificial sweeteners (aspartame, acesulfame potassium, saccharin, sucralose) and stevia, the natural sweetener; synthetic food dyes (Blue 1, Blue 2, Green 3, Red 3, Red 40, Yellow 5, Yellow 6); and other selected additives (titanium dioxide and TBHQ). 

### 2.3. Statistical Analyses

Descriptive statistics—including school characteristics, school mealtime policies, and participation in national programs such as CEP—were calculated for each school. The availability of competitive foods was calculated as the number of products per school. Percentage compliance with the Smart Snacks standards—overall and by the individual nutrient standards—was calculated for unique products as well as by product type. Alignment with the DGA for added sugars was also calculated as a percentage of calories from added sugar for each product [9,28].

Mixed methods analysis of variance was used to examine differences in compliance with the Smart Snacks standards (overall and by product type) and comparing them according to school characteristics, including: school level (middle or high school), FNS region, urbanicity, who oversaw the operation of competitive foods, and the location of the competitive foods. Adjusted average compliance with the Smart Snacks standards was estimated using least squares mean regression. Other covariates examined include: the percentage of students eligible for FRPM, whether school meals were free for all students (either through CEP or UFSM), and open campus policies. However, these were not statistically significant and, therefore, were not included in the final models. In a sensitivity analysis, New Jersey was excluded, but there were no meaningful differences in the study results and the state was included in the final analyses. Analyses were conducted using SAS statistical software (version 9.4, SAS Institute Inc., Cary, NC, USA). Results were considered statistically significant if the *p*-value was <0.05 (2-sided). 

## 3. Results

### 3.1. School Characteristics

Among the participating districts, student eligibility for FRPM ranged from less than 25% to greater than 60% eligibility (Table 1). Free school meals were available to all students through CEP or state-level UFSM policies in 38% of the middle schools and 40% of the high schools participating in the study. Slightly under half of the high schools (44%) had open-campus policies, while only 7% of middle schools had these policies. 

Overall, the majority of schools (80%; *n* = 72 schools) sold competitive foods, and 87% of high schools and 73% of middle schools sold these snacks and beverages to students, most commonly in à la carte lines and vending machines. Among all the high schools in the study, 82% sold beverages, salty snacks, and sweet snacks; 42% sold ice cream or frozen treats; 9% sold yogurt or cheese; and 4% (two high schools) sold fruits or vegetables as competitive foods. Across all the middle schools, 60% sold beverages, 58% sold salty snacks, 53% sold sweet snacks, 33% sold ice cream or frozen treats, 9% sold yogurt or cheese, and 2% (one middle school) sold fruits or vegetables. Slightly less than 10% of both high schools (*n* = 4) and middle schools (*n* = 4) had school stores with snacks and beverages. Among the schools that did not sell competitive foods, roughly two-thirds (65%) provided free school meals to all students, and roughly half participated in CEP and half participated through state-level UFSM policies.

In the high schools that sold competitive foods there was, on average, a total of 4.3 locations (median = 4; range 1–13) per school; this includes, on average, 3.4 vending machines (median = 3; range 0–13). There were fewer locations on average in the middle schools that sold competitive foods; there were two locations on average (median = 1; range 1–6), including, on average, 1.1 vending machines (median = 0; range 0–5). Among both the middle and high schools with vending machines, 61% of food service directors reported that vending machines were overseen by someone outside of the food service department (e.g., by outside vending companies, principals, student organizations, athletics clubs, parent-teacher organization [PTO], etc.). 

### 3.2. Availability and Compliance among Unique Competitive Foods

Overall, a total of 840 unique competitive foods were observed across the school sites (Table 2), and 58% of these products were compliant with all of the Smart Snacks standards. This included *n* = 5 Smart Snacks-compliant fruit and vegetables (including combination options with dips or nut butters [100% compliant]); *n* = 9 yogurt and cheese products (89% compliant); *n* = 100 ice creams and frozen treats (78% compliant); *n* = 369 beverages (69% compliant); *n* = 208 sweet snacks (e.g., cookies, granola bars, pastries, candy [43% compliant]); and *n* = 149 salty snacks (e.g., chips, crackers, pretzels [41% compliant]). Compliance was higher for unflavored and flavored water (100% compliance), 100% fruit juice (94%), and milk products (82%); and was lower for low- and no-calorie beverages (41% [Supplementary Table S1]).

### 3.3. Competitive Food Offerings and Compliance within Schools

When examining the entire school environment, in which the same foods could be served in multiple locations (vending machines, snack carts, etc. [i.e., “non-unique” products offered throughout the schools]), there were, on average, 54 snacks and beverages offered per high school and 24 snacks and beverages offered per middle school (Table 3). High schools offered, on average, 22 beverages, 16 salty snacks, 13 sweet snacks, three ice creams or frozen treats, and fewer than one yogurt/cheese, or fruit/vegetable. Middle schools offered, on average, six beverages, nine salty snacks, six sweet snacks, three ice creams or frozen treats, and fewer than one yogurt/cheese, or fruit/vegetable. The nutrient profile of the overall (non-unique) competitive foods offered across all locations is presented in Supplementary Table S2. 

**Figure 1:**
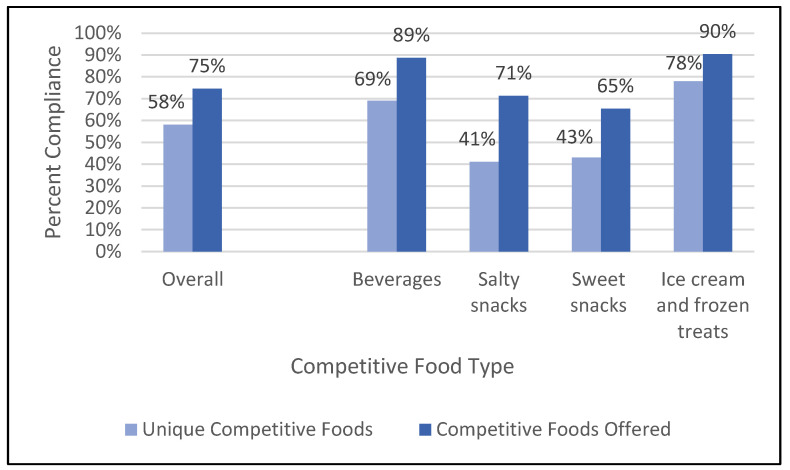
Competitive food compliance with the Smart Snacks standards in a national sample of middle and high schools (Among *n* = 72 middle and high schools in a national sample of schools selling competitive foods).

Compliance among all (i.e., non-unique) products offered throughout the schools was higher compared with that for unique products because compliant competitive foods were offered more frequently (Figure 1). Overall, 74% of the non-unique competitive foods in high schools were in alignment with the standards, and 76% were in alignment in middle schools. There was high compliance across all competitive food categories (Table 3). Compliance rates by food type among non-unique products did not differ substantially between middle and high schools. Overall, only four schools (6% of the schools selling competitive foods) were in full compliance with the Smart Snacks standards (i.e., all competitive foods were compliant), and 18 schools (25% of the schools) were at least 90% in compliance with the standards.

In multivariate analyses, there was greater compliance with Smart Snacks standards among competitive foods overseen by food service departments compared with competitive foods overseen by others such as principals, outside vending companies, or student organizations (77% vs. 59% compliance; *p* = 0.003 [Table 4]). There was also greater compliance among competitive foods sold inside cafeterias compared with all other locations; the lowest compliance was in school stores (37% average compliance in school stores compared with 87% inside cafeterias; *p* < 0.001). Some regional differences were also observed. For example, there was lower compliance in the mid-Atlantic regions compared with other regions. There were no significant differences by urbanicity or between middle and high schools. 

Similar trends in the predictors of overall compliance were observed in the multivariate analysis by product category for beverages, salty snacks, and sweet snacks (Supplementary Table S3). No differences were observed between school characteristics when examining compliance for ice cream and frozen treats (and there were too few yogurt/cheese products and fruits/vegetable products to examine differences by school characteristics). 

Examining each component of the Smart Snacks standards separately (Supplementary Table S4), snacks overall—as well as the subcategories of both salty snacks and sweet snacks—were, on average, in greater alignment with the calorie, saturated fat, sodium, sugar, and other (e.g., whole grain) standards when the competitive foods were sold in the cafeteria compared with in a school store or other location. They were also in greater alignment with the calorie, saturated fat, and sodium standards when the food service director oversaw the competitive foods.

### 3.4. Added Sugars

Among the unique competitive foods, average added sugars, which are not at present an element of the Smart Snacks standards, ranged from 0% of calories (fruits and vegetables [median = 0; range 0–0) to 37% of calories for beverages (median = 0; range 0–100%) (Table 2). Overall, 58% of the competitive foods had less than 10% of calories from added sugar, including 73% of beverages, 87% of salty snacks, 21% of sweet snacks, 36% of ice cream and frozen treats, 44% of yogurts and cheeses, and 100% of fruits and vegetables. A total of 40% of unique products (*n* = 340) were both Smart Snacks standards-compliant and in alignment with DGA recommended limits for added sugar. This included 64% of beverages, 33% of salty snacks, 12% of sweet snacks, 25% of ice cream and frozen treats, 33% of yogurt and cheese products, and 100% of fruit and vegetable products. Similar results were observed among non-unique products offered across multiple locations throughout the schools. 

### 3.5. Non-Sugar Sweeteners, Synthetic Dyes, and Other Additives

Overall, 16% of the unique competitive foods contained non-sugar sweeteners (including both artificial and natural sweeteners) and 21% contained synthetic dyes (Table 2). Specifically, among beverages, 35% contained non-sugar sweeteners (31% of beverages contained artificial sweeteners when excluding the natural sweetener, stevia). Among products with artificial sweeteners, sucralose was the most common, occurring in 64% of products, and nearly a third had aspartame (30%) or acesulfame potassium (32%); no products contained saccharin. A total of 10% of products containing non-sugar sweeteners contained stevia. When examining non-sugar sweeteners in the non-unique competitive foods offered across multiple locations throughout the schools—which the Smart Snacks standards allow in high schools but not in middle schools—over half of the beverages in high schools had non-sugar sweeteners (47% contained artificial sweeteners when excluding the natural sweetener, stevia); and 14% of beverages in middle school had artificial sweeteners (none contained stevia [Supplementary Table S2]). Non-sugar sweeteners were rare in sweet snacks and absent in salty snacks, ice cream and frozen treats, yogurt and cheese, and fruits and vegetables in the middle or high schools.

When examining synthetic dyes among unique products, overall 20% of beverages, 20% of salty snacks, 22% of sweet snacks, and 29% of ice creams and frozen treats contained synthetic dyes. Among the products containing synthetic dyes, the most common were Red No. 40 (62% of products), Blue No. 1 (50%), Yellow No. 5 (44%), and Yellow No. 6 (36%). Only 2% of unique competitive food products contained titanium dioxide and 1% contained TBHQ. Among the non-unique competitive foods offered across multiple locations throughout the schools, 25% of beverages in high schools and 9% of those sold in middle schools contained synthetic dyes. About a third of salty snacks, and ice cream and frozen treats in both middle school and high school contained synthetic dyes. Among sweet snacks, 17% of high-school and 10% of middle-school products contained synthetic dyes. None of the yogurts and cheese, or fruits and vegetables contained synthetic dyes. 

## 4. Discussion

This national study found that over 70% of middle schools and nearly 90% of high schools sold snacks and beverages to students outside of reimbursable school meals. Competitive foods were more common in schools that did not provide free school meals to all students through CEP or state-level UFSM policies. More than 800 unique competitive foods were observed across the participating schools. Beverages, salty snacks, sweet snacks, and ice cream and frozen treats were the most commonly offered; and a greater number of products were available, on average, in high schools. Most products (58%) were compliant with Smart Snacks standards, and compliant products were more likely to be sold at multiple locations within schools such that roughly 75% of the non-unique competitive foods offered were in alignment with those standards. The lowest compliance was observed for salty snacks (41%) and sweet snacks (43%). Compliance was significantly greater when competitive foods were overseen by food service departments rather than principals, student organizations, or other groups. A total of 35% of beverages contained non-sugar sweeteners; the majority contained artificial sweeteners. Roughly one-fifth of beverages, salty snacks, sweet snacks, and ice creams and frozen treats contained synthetic dyes. Over half of the competitive foods observed were in alignment with DGA recommendations for added sugar. While slightly less than 10% of schools had school stores, the competitive foods sold there were, on average, less likely to be compliant than competitive foods sold elsewhere.

The overall gaps in compliance with the Smart Snacks standards observed in the present study were similar to those found by the national School Nutrition and Meal Cost Study, which was conducted during the 2014–2015 school year, soon after implementation of the Smart Snacks standards [9,28]. Similar levels of compliance were also observed in an observational cohort study (the NOURISH study) that examined a very similar Massachusetts competitive food law prior to implementation of the national Smart Snacks standards [9,28]. Therefore, the results of the present study also suggest that, in nearly a decade, compliance with the Smart Snacks standards has not increased further. This highlights the need for improved implementation and enforcement of the standards. Importantly, the NOURISH study also found that implementing the healthier competitive food standards was associated with improvements in students’ diets, including reductions in students’ total sugar intake [30]. While both the present study and the NOURISH study found fewer products for sale to students in middle schools compared with high schools, this study found that overall compliance did not differ by grade level, whereas the NOURISH study found greater compliance among middle schools [9]. 

The results of this study suggest that both a DGA-consistent standard for added sugars is feasible and that there is substantial room for improvement. The present study documented hundreds of products already available that are in alignment with both the Smart Snacks standards and DGA recommendations; this supports the feasibility of implementing limits on added sugars for competitive foods. The compliance levels observed in the present study would be even higher if an added sugar standard was based on gram limits (e.g., ≤5 g of added sugars for snacks with 200 kcal or less), rather than as a percentage of calories; this further highlights the feasibility of limits on added sugars. Restrictions on the presence of artificial sweeteners should also be considered to prevent unintended consequences related to restrictions on added sugars. Encouragingly, 40% of beverages in the present study were not only compliant with the current Smart Snack standards, but also contained less than 10% of calories from added sugar and did not have artificial sweeteners. Similarly, there was a substantial percentage of competitive foods with synthetic dyes, but also many options without; so future competitive food standards should consider restrictions on synthetic dyes associated with adverse health outcomes [20]. Future research should also examine the levels of caffeine in the competitive beverages available.

The primary limitation of this study was the school district participation rate and representativeness; only roughly one-third of school districts that were randomly selected agreed to be in the study. However, this participation rate is commonly observed in school-based nutrition research, including prior research examining competitive foods in schools; time constraints are commonly reported by food service directors as a major limiting factor when recruiting schools [9,28]. It is, nonetheless, quite possible that there was selection bias among participating school districts. Districts that declined were perhaps more likely to sell non-compliant competitive foods. This would result in the present study overestimating compliance, which further highlights the need to improve alignment with the federal competitive food standards. It is reassuring, therefore, that the districts that declined to participate were similar regarding school characteristics—including the percentage of students eligible for FRPM and free school meal policies—to those that enrolled in the study. Second, fundraisers—which are another avenue for the sale of competitive foods—were not examined, and future research should evaluate how frequently foods from fundraisers, as well as foods at athletic events and other extracurricular activities, are sold to students. Similarly, foods and beverages that were provided to students as part of the school breakfast or lunch program were not included in the present study if they were not also listed by the school as a competitive food. While it Is possible that some of these meal items may have been sold to students individually, it was likely a negligible number, given that they were not visibly promoted by the school as competitive foods (nor did the school have individual prices for these meal items). The strengths of this study are its sample of geographically and socioeconomically diverse school districts, and the rigorous methods of direct observations to assess the presence of competitive foods. This was also the first comprehensive national study to examine competitive foods since the national School Nutrition and Meal Cost Study was conducted nearly a decade ago.

## 5. Conclusions

This national study of competitive foods found that most middle and high schools sold competitive foods, the majority of which were compliant with the Smart Snacks standards. Further, a substantial number of products sold in schools were in alignment with stronger standards; such as those for limits on added sugars and other additives. Lower compliance was observed in school stores and with competitive foods not overseen by food service departments. Therefore, school districts should consider requiring food service departments to oversee snacks and beverages in school stores. Similar policies could also be considered at the state and federal levels. Additionally, a comprehensive national database with compliant competitive foods, similar to state level-initiatives like the John C. Stalker Institute competitive food database in Massachusetts, could help schools identify compliant products and further improve alignment with the standards. The results of this study also strongly suggest that the USDA could feasibly establish science-based limits on added sugars in competitive foods, as well as greater restrictions on artificial sweeteners. As nearly a fifth of the competitive foods in this study contained synthetic dyes, limits on these additives should also be considered, as well as future revisions to the Smart Snacks standards. Lastly, competitive food sales were less prevalent in schools where school meals were free to all students; therefore, UFSM policies should be considered to support healthier school meal environments. 

## Figures and Tables

**Table 1:** Characteristics of a national sample of *n* = 90 middle and high schools ^1^.

Characteristics	Middle (*n* = 45)	High (*n* = 45)
Eligibility for FRPM, number (%)		
≤24%	9 (20%)	8 (18%)
25–40%	16 (36%)	17 (38%)
41–59%	9 (20%)	9 (20%)
≥60%	11 (23%)	11 (23%)
Free school meals (CEP or state UFSM policy), number (%)	17 (38%)	18 (40%)
Open campus policy ^2^, number (%)	3 (7%)	20 (44%)
Schools with competitive foods, number (%)	33 (73%)	39 (87%)
Schools with à la carte lines, number (%)	25 (56%)	31 (69%)
Schools with school stores, number (%)	4 (9%)	4 (9%)
Schools with vending machines, number (%)	17 (38%)	31 (69%)
Total vending machines per school, average (range) ^1^	1.1 (0–5)	3.4 (0–13)
Total competitive food locations, average (range) ^1^	2.0 (1–6)	4.3 (1–13)

CEP = Community Eligibility Provision. FRPM = free or reduced-price meal. UFSM = Universal Free School Meal. ^1^ Among the subsample of schools (*n* = 72) with competitive foods. ^2^ Open campus policies enable some students (e.g., grade 12 only) or all students within a school to leave during lunch.

**Table 2:** Average Smart Snacks standards compliance, nutrients, and additives among *n* = 840 unique competitive foods observed in a national sample of middle and high schools ^1^.

Category	Smart Snacks Standards-Compliant (%)	Calories (kcal) *, Range	Total Fat (g), Range	Total Fat (%) *, Range	Saturated Fat (g), Range	Saturated Fat (%) *, Range	Trans Fat (g) *, Range	Sodium (mg) *, Range	Total Sugar (g) *, Range	Added Sugar (g), Range	Percentage Calories from Added Sugar, Range	Fiber (g), Range	Contains Non- Sugar Sweeteners ^2^* (%)	Contains Synthetic Dye(s) ^3^ (%)
Overall (*n* = 840)	58%	118 (0–720)	3.1 (0–34)	4.0 (0–45)	0.9 (0–16)	4.2 (0–45)	0.0 (0)	108 (0–960)	12.9 (0–102)	9.0 (0–102)	28% (0–100%)	0.7 (0–12)	16%	21%
Beverages (*n* = 369)	69% ^4^	62 (0–290)	0.1 (0–4.5)	0.1 (0–6)	0.0 (0–2.5)	0.2 (0–13)	0.0 (0)	66 (0–960)	14.0 (0–73)	9.0 (0–73)	37% (0–100%)	0.1 (0–5)	35% ^6^	20%
Salty snacks (*n* = 149)	41%	158 (60–450)	7.5 (0.5–20)	9.9 (1–45)	1.3 (0–9)	6.6 (0–45)	0.0 (0)	217 (25–900)	2.2 (0–20)	1.5 (0–16)	4% (0–32%)	1.4 (0–12)	0%	19%
Sweet snacks (*n* = 208)	43%	188 (0–720)	5.7 (0–34)	7.6 (0–45)	1.9 (0–16)	9.3 (0–26)	0.0 (0)	132 (0–620)	16.7 (0–102)	13.3 (0–102)	32% (5–96%)	1.6 (0–9)	2%	22%
Ice cream and frozen treats (*n* = 100)	78%	123 (40–320)	2.2 (0–12)	2.5 (0–15)	1.1 (0–7)	5.1 (0–35)	0.0 (0)	59 (0–210)	17.5 (6–37)	9.2 (0–37)	34% (5–87%)	0.2 (0–2)	0%	29%
Yogurt and cheese (*n* = 9)	89%	110 (60–220)	2.8 (0–10)	3.7 (0–13)	1.3 (0–4)	6.6 (0–20)	0.0 (0)	121 (45–310)	9.8 (0–23)	5.3 (0–17)	18% (0–38%)	0.4 (0–3)	0%	0%
Fruits and vegetables ^5^ (*n* = 5)	100%	98 (30–260)	3.2 (0–16)	5.0 (0–25)	0.6 (0–3)	3.0 (0–15)	0.0 (0)	29 (0–140)	12.2 (6–16)	0.0 (0–0)	0% (0–0%)	2.4 (1–5)	0%	0%

* Regulated by the Smart Snacks standards (total sugar is regulated as a percentage of the product weight [≤35% by weight]). ^1^ Among *n* = 72 middle and high schools in a national sample of schools selling competitive foods. ^2^ Non-sugar sweeteners (i.e., artificial sweeteners and other sugar substitutes) are allowed in beverages by the Smart Snacks standards within high schools only. Those examined included the natural sweetener stevia, and the artificial sweeteners: aspartame (e.g., NutraSweet and Equal); acesulfame potassium (e.g., Sweet One); saccharin (e.g., Sweet’N Low); and sucralose (e.g., Splenda). ^3^ Synthetic food dyes and other additives are not regulated by the Smart Snacks standards. Those examined included: Blue No. 1; Blue No. 2; Green No. 3; Red No. 3; Red No. 40; Yellow No. 5; Yellow No. 6; titanium dioxide; and tert-butylhydroquinone (TBHQ). ^4^ Includes products that are considered compliant only among high schools, but would not be compliant if offered in a middle school. ^5^ Includes combinations of fruit or vegetables with dips or nut butters (e.g., apples with peanut butter). ^6^ A total of 31% of products contained artificial sweeteners (i.e., excluding the natural sweetener, stevia). Unique competitive snack foods were most commonly non-compliant due to exceeding the requirements for total calories, saturated fats, total sugar, or sodium; as well as grain-based products not being at least 50% whole grain (Supplementary Table S1). Specifically, among beverages that were non-compliant with the Smart Snacks standards, the primary reasons were products exceeding calorie limits (60 kcals per 12 fluid ounces) or exceeding portion size limits. The primary reason for non-compliance among both salty snacks and sweet snacks was due to not meeting the whole-grain requirement (59% and 57% non-compliance among products, respectively). The primary reason ice cream and frozen treats did not meet the Smart Snacks standards was due to saturated fat levels exceeding the standards (22% of products).

**Table 3:** Smart Snacks standards compliance and availability among *n* = 2824 competitive foods offerings in a national sample of middle and high schools ^1^.

Category	Middle School	High School
All foods	*n* = 779	*n* = 2045
Total number compliant	592	1513
% Compliant ^2^	76%	74%
Average number per school ^3^	23.7	53.8
Beverages	*n* = 181	*n* = 836
Total number compliant	136	677
% Compliant	75%	81%
Average number per school	6.1	22.0
Salty snacks	*n* = 295	*n* = 610
Total number compliant	218	427
% Compliant	74%	70%
Average number per school	8.6	16.1
Sweet snacks	*n* = 184	*n* = 481
Total number compliant	142	293
% Compliant	77%	61%
Average number per school	5.5	12.7
Ice cream and frozen treats	*n* = 113	*n* = 106
Total number compliant	97	101
% Compliant	86%	95%
Average number per school	3.1	2.8
Yogurt and cheese	*n* = 4	*n* = 6
Total number compliant	4	5
% Compliant	100%	83%
Average number per school	0.4	0.2
Fruits and vegetables ^4^	*n* = 2	*n* = 6
Total number compliant	2	6
% Compliant	100%	100%
Average number per school	0.1	0.2

^1^ Among *n* = 72 middle and high schools in a national sample of schools selling competitive foods, which includes the total number of products available across multiple locations within a school (i.e., non-unique products). ^2^ % Compliant = number of Smart Snacks standards-compliant competitive foods/total number of competitive foods. Compliance only calculated among schools that offered any competitive foods of items available per school. ^3^ Calculated as the total number of items available per location per school (e.g., if the same product is available in four vending machines, on an à la carte line, and in a school store, this counts as available six times). Availability is averaged across all participating schools. ^4^ Includes combinations of fruit or vegetables with dips or nut butters (e.g., apples with peanut butter).

**Table 4:** Differences in Smart Snacks standards compliance by school characteristics among *n* = 2824 competitive foods available in a national sample of middle and high schools ^1^.

	% Compliance ^2^	* p * -Value ^3^
Grade level		
High	74%	Ref
Middle	76%	0.6
Urbanicity		
Urban	65%	Ref
Suburban	68%	0.7
Rural	71%	0.3
FNS region		
Mid-Atlantic	48%	Ref
Midwest	67%	0.02
Mountain Plains	74%	0.007
Northeast	71%	0.009
Southeast	81%	0.005
Southwest	78%	0.0002
West	58%	0.3
Location		
Inside cafeteria	87%	Ref
School store	37%	<0.0001
Other locations	70%	<0.0001
Oversees vending machines		
Food Service Directors	73%	Ref
Other ^4^	56%	0.004

^1^ Among *n* = 72 middle and high schools in a national sample of schools selling competitive foods. ^2^ % compliant = number of Smart Snacks standards-compliant competitive foods/total number of competitive foods. Compliance is only calculated among schools that offered any competitive foods. Average compliance is estimated using least squares means regression. ^3^ Calculated using mixed methods analysis of variance, accounting for repeated measures within schools and clustering within school districts (all covariates simultaneously included in models). ^4^ Others include outside vendors, principal, student organizations, or other organizations.

## Data Availability

The data presented in this study are available on request from the corresponding author. The data are not publicly available due to additional analyses currently being conducted with the dataset, and therefore to prevent inadvertent duplication of research [insert reason here].

## Supplementary Materials

The following supporting information can be downloaded at: https://www.mdpi.com/article/10.3390/nu16020275/s1, Figure S1: States included in competitive food study by United States Department of Agriculture’s Food and Nutrition Service Administrative Regions; Table S1: Percent compliance with each component of the Smart Snacks standards among *n* = 840 unique competitive foods observed in a national sample of middle and high schools; Table S2: Average Smart Snacks compliance, nutrients, and artificial sugar and dyes among *n* = 2824 competitive foods offered in a national sample of middle and high schools with competitive foods; Table S3: Differences in Smart Snacks compliance among different competitive food categories by school characteristics; Table S4: Percent compliance with the Smart Snacks standards by nutrient standard among competitive foods observed in a national sample of middle and high schools.
