# Supplementary material for: Competitive Foods’ Nutritional Quality and Compliance with Smart Snacks Standards: An Analysis of a National Sample of U.S. Middle and High Schools
Source: Nutrients. 2024 Jan 17;16(2):275. doi: 10.3390/nu16020275 (PMC10818474; doi:10.3390/nu16020275)
Supplement: Supplementary file 1 [file nutrients-16-00275-s001.zip › nutrients-2798196-supplementary.pdf]

Supplemental Figure 1. States included in competitive food study by United States Department of Agriculture's Food and Nutrition Service Administrative Regions

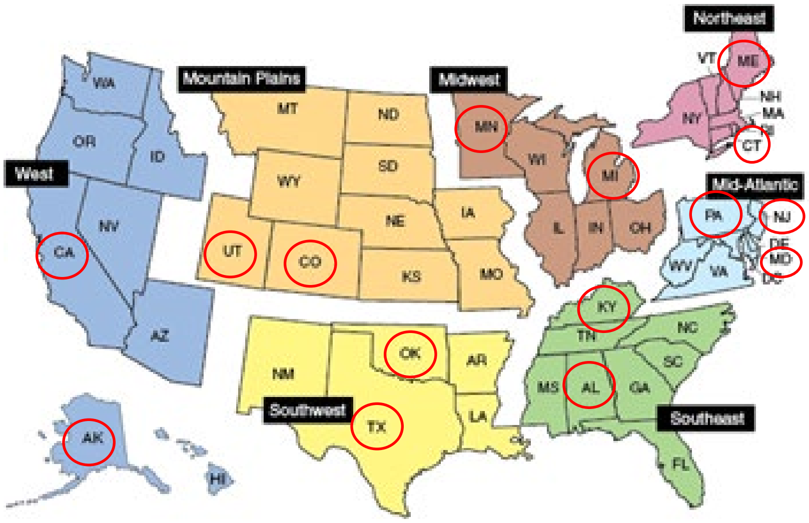

Supplemental Table 1. Percent compliance with each component of the Smart Snacks standards among n=840 unique competitive foods observed in a national sample of middle and high schools[1]

Category	Overall Smart Snacks compliance (%)	Calories (≤200 kcal)	Percent of calories from total fat (≤35%)	Percent of calories from saturated fat (<10%)	Trans fat (0g)	Sodium (≤200 mg)	Total sugar (≤ 35% by weight)	Other[2]
Beverages (n=369)								
Water: unflavored (n=22)	100%	N/A	N/A	N/A	N/A	N/A	N/A	N/A
Water: flavored (n=117)	100%*	N/A	N/A	N/A	N/A	N/A	N/A	N/A
Milk products[3] (n=11)	82%	N/A	N/A	N/A	N/A	N/A	N/A	N/A
100% fruit juice[4] (n=33)	94%	N/A	N/A	N/A	N/A	N/A	N/A	N/A
Low and no-calorie beverages[5] (n=186)	41%*	N/A	N/A	N/A	N/A	N/A	N/A	N/A
Snacks (n=471)								
Salty snacks (n=149)	41%	75%	99%	81%	100%	63%	99%	41%
Sweet snacks (n=208)	43%	69%	99%	72%	100%	80%	68%	43%
Ice cream and frozen treats (n=100)	78%	97%	100%	78%	100%	99%	96%	94%
Yogurt and cheese (n=9)	89%	89%	100%	89%	100%	89%	89%	100%
Fruits and vegetables[6] (n=5)	100%	100%	100%	100%	100%	100%	100%	100%

* Includes products with non-sugar sweeteners (i.e., artificial sweeteners and other sugar substitutes) that are considered compliant only among high schools, but would not be compliant if offered in a middle school.

[1] Among n= 72 middle and high schools in a national sample of schools selling competitive foods.

[2] "Other" includes: (1) a grain product that contains 50 percent or more whole grains by weight (have a whole grain as the first ingredient); (2) a product with the first ingredient being a fruit, a vegetable, a dairy food, or a protein food; or (3) a combination food that contains at least ¼ cup of fruit and/or vegetable

[3] Milk includes unflavored or flavored fat-free milk, low-fat milk, and milk alternatives as permitted by the National School Lunch Program and School Breakfast program. Compliance for milk products is ≤ 12 fluid ounces.

[4] Compliance for 100% fruit juice is ≤ 12 fluid ounces.

[5] Low and no-calorie beverages (high school only) include low calorie drinks with the equivalent of 5 calories per fluid ounce (maximum of 60 kcals/12 fluid ounces) and no-calorie beverages with less than 5 calories per 8 fluid ounces (maximum of 10 calories/20 fluid ounce).

[6] Includes combinations of fruit or vegetables with dips or nut butters (e.g., apples with peanut butter).

Supplemental Table 2. Average Smart Snacks compliance, nutrients, and artificial sugar and dyes among n=2,824 competitive foods offered in a national sample of middle and high schools with competitive foods[1]

Category	Smart Snacks compliant (%)	Calories (kcal)*, range	Total fat (g), range	Total fat (%)*, range	Saturated fat (g), range	Saturated fat (%)*, range	Sodium (mg)*, range	Total sugars (g)*, range	Added sugars (g), range	Percent calories from added sugars	Fiber (g), range	Contains non- sugar sweeteners[2]* (%)	Contains synthetic dye(s)[3] (%)
Beverages													
Middle (n=181)	75%	75 (0-290)	0.0 (0-4.5)	0.0 (0-6)	0.0 (0-0.5)	0.0 (0-3)	39 (0-270)	16.9 (0-73)	2.9 (0-73)	15% (0-100%)	0.0 (0-2)	12%	8%
High (n=836)	81%	37 (0-290)	0.0 (0-4.5)	0.0 (0-6)	0.0 (0-2.5)	0.1 (0-13)	79 (0-960)	8.1 (0-73)	5.1 (0-73)	27% (0-100%)	0.0 (0-5)	52%[5]	25%
Salty Snacks													
Middle (n=295)	74%	138 (60-330)	5.6 (0.5-29)	7.5 (1-45)	0.8 (0-4)	4.3 (0-21)	201 (65-900)	1.5 (0-16)	1.0 (0-16)	2.9% (0-29%)	1.6 (0-12)	0%	32%
High (n=610)	70%	140 (60-450)	5.8 (1-29)	7.7 (1-45)	0.9 (0-9)	4.7 (0-45)	195 (25-520)	1.7 (0-20)	1.2 (0-16)	3.5% (0-32%)	1.5 (0-12)	0%	30%
Sweet Snacks													
Middle (n=184)	77%	166 (50-400)	4.4 (0-14)	5.8 (0-36)	1.2 (0-6)	6.0 (0-23)	133 (0-620)	13.3 (2-52)	12.2 (0-32)	30% (0-74%)	1.9 (0-9)	0%	10%
High (n=481)	61%	182 (0-720)	5.2 (0-34)	6.8 (0-45)	1.7 (0-16)	8.3 (0-26)	125 (25-540)	15.8 (0-102)	14.7 (0-102)	32% (0-96%)	1.7 (0-9)	1%	17%
Ice Cream and Frozen Treats													
Middle (n=113)	86%	127 (40-320)	2.5 (0-12)	2.9 (0-15)	1.1 (0-7)	5.2 (0-35)	61 (0-210)	16.7 (6-37)	10.1 (0-37)	34% (0-82%)	0.2 (0-1)	0%	33%
High (n=106)	95%	122 (60-200)	2.3 (0-12)	3.0 (0-15)	1.0 (0-7)	5.3 (0-35)	59 (0-170)	15.5 (11-30)	9.4 (0-17)	33% (0-68%)	0.2 (0-2)	0%	38%
Yogurt and Cheese													
Middle (n=4)	100%	83 (60-100)	0.0 (0-0)	0.0 (0-0)	0.0 (0-0)	0.0 (0-0)	58 (45-70)	10.5 (9-12)	5.0 (4-6)	25% (20-29%)	0.0 (0-0)	0%	0%
High (n=6)	83%	140 (80-220)	4.5 (0-10)	6.0 (0-13)	2.2 (0-4)	10.7 (0-20)	159 (75-319)	11.5 (0-23)	7.8 (0-17)	17% (0-38%)	0.8 (0-3)	0%	0%
Fruits and Vegetables[4]													
Middle (n=2)	100%	145 (30-260)	8.0 (0-16)	12.5 (0-25)	1.5 (0-3)	7.5 (0-15)	70 (0-140)	10.5 (6-15)	0.0 (0-0)	0% (0-0)	3.0 (1-5)	0%	0%
High (n=6)	100%	125 (30-260)	5.3 (0-16)	8.3 (0-25)	1.0 (0-3)	5.0 (0-15)	48 (0-140)	12.7 (6-16)	0.0 (0-0)	0% (0-0)	2.8 (1-5)	0%	0%

*Regulated by the Smart Snack standards (total sugar is regulated as a percentage of the product weight [≤ 35% by weight])

[1] Among n= 72 middle and high schools in a national sample of schools selling competitive foods, which includes the total number of products available across multiple locations within a school.

[2]Non-sugar sweeteners (i.e., artificial sweeteners and other sugar substitutes) are allowed by the Smart Snacks standards within high schools only. Those examined included: aspartame (e.g., NutraSweet and Equal); acesulfame potassium (e.g., Sweet One); saccharin (e.g., Sweet'N Low); sucralose (e.g., Splenda); and stevia.

[3] Synthetic food dyes and other additives are not regulated by the Smart Snacks standards. Those examined included: Blue No. 1; Blue No. 2; Green No. 3; Red No. 3; Red No. 40; Yellow No. 5; Yellow No. 6; titanium dioxide; and tert-butylhydroquinone (TBHQ).

[4] Includes combinations of fruit or vegetables with dips or nut butters (e.g., apples with peanut butter)

[5] 47% of products contained artificial sweeteners in high schools (i.e., excluding the natural sweetener stevia). Stevia was not present in any beverages in middle schools nor in any food products.

Supplemental Table 3. Differences in Smart Snacks compliance among different competitive food categories by school characteristics[1]

Category	% Compliance [2]	P-value[3]
Beverages (n=1,017)		
Grade Level		
High	76%	Ref*
Middle	73%	0.6
Urbanicity		
Urban	68%	Ref
Suburban	76%	0.2
Rural	79%	0.1
FNS Region		
Mid Atlantic	52%	Ref
Midwest	76%	0.02
Mountain Plains	77%	0.05
Northeast	79%	0.02
Southeast	76%	0.2
Southwest	87%	0.001
West	74%	0.1
Location		
Inside Cafeteria	86%	Ref
School Store	62%	0.002
Other Locations	75%	0.005
Oversees Competitive Foods		
Food Service Director	80%	Ref
Other[4]	69%	0.1
Salty Snacks (n=905)		
Grade Level		
High	54%	Ref
Middle	58%	0.4
Urbanicity		
Urban	58%	Ref
Suburban	55%	0.8
Rural	55%	0.8
FNS Region		
Mid Atlantic	43%	Ref
Midwest	52%	0.6
Mountain Plains	64%	0.2
Northeast	48%	0.7
Southeast	74%	0.1
Southwest	68%	0.1
West	41%	0.9
Location		
Inside Cafeteria	87%	Ref
School Store	16%	<0.0001
Other Locations	65%	<0.0001
Oversees Competitive Foods		
Food Service	67%	Ref

Other	45%	0.04
Sweet Snacks (n=665)		
Grade Level		
High	56%	Ref
Middle	63%	0.2
Urbanicity		
Urban	60%	Ref
Suburban	59%	0.9
Rural	60%	0.9
FNS Region		
Mid Atlantic	43%	Ref
Midwest	54%	0.4
Mountain Plains	70%	0.1
Northeast	68%	0.1
Southeast	80%	0.06
Southwest	67%	0.08
West	35%	0.7
Location		
Inside Cafeteria	84%	Ref
School Store	32%	<0.0001
Other Locations	63%	<0.0001
Oversees Vending Machines		
Food Service	70%	Ref
Other	49%	0.04

*Reference category.

[1] Among n= 72 middle and high schools in a national sample of schools selling competitive foods (n=2,824 competitive foods offered across multiple locations within the schools)

[2] % Compliant = number of Smart Snacks compliant competitive foods / total number of competitive foods. Compliance is estimated using least squares means regression.

[3] Calculated using mixed methods analysis of variance, accounting for repeated measures within schools and clustering within school districts (all covariates simultaneously included in models).

[4] Other includes outside vendors, principal, student organizations, or other organizations.

Supplemental Table 4. Percent compliance with the Smart Snacks standards by nutrient standard among competitive foods observed in a national sample of middle and high schools[1]

All Snacks										
	Calories (≤200 kcal)		Percent of calories from saturated fat (<10%)		Sodium (≤200 mg)		Total sugar (≤ 35% by weight)		Other[2]	
	% Compliance [2]	P-value[3]	% Compliance	P-value	% Compliance	P-value	% Compliance	P-value	% Compliance	P-value
Urbanicity										
Urban	83%	Ref	81%	Ref	83%	Ref	87%	Ref	57%	Ref
Suburban	84%	0.9	89%	0.6	82%	0.8	84%	0.3	58%	0.9
Rural	91%	0.1	84%	0.1	87%	0.5	81%	0.1	61%	0.6
FNS Region										
Mid Atlantic	79%	Ref	71%	Ref	79%	Ref	85%	Ref	42%	Ref
Midwest	84%	0.5	83%	0.1	85%	0.5	78%	0.2	57%	0.2
Mountain Plains	97%	0.05	87%	0.06	93%	0.1	84%	0.8	67%	0.05
Northeast	90%	0.2	85%	0.07	87%	0.4	87%	0.7	58%	0.2
Southeast	94%	0.2	97%	0.01	88%	0.4	88%	0.8	77%	0.03
Southwest	95%	0.04	86%	0.03	94%	0.05	85%	0.9	70%	0.01
West	64%	0.1	84%	0.1	64%	0.1	80%	0.4	41%	0.9
Location										
Inside Cafeteria	87%	Ref	95%	Ref	92%	Ref	99%	Ref	85%	Ref
School Store	87%	0.2	83%	0.0001	82%	0.04	65%	<0.0001	30%	<0.0001
Other Locations	76%	<0.0001	76%	<0.0001	79%	<0.0001	88%	<0.0001	62%	<0.0001
Oversees Vending Machines										
Food Service	93%	Ref	91%	Ref	90%	Ref	85%	Ref	69%	Ref
Other	79%	0.03	79%	0.02	79%	0.04	83%	0.7	49%	0.01
Type of Snack										
Salty snacks (n=905)										
Urbanicity										
Urban	80%	Ref	83%	Ref	73%	Ref	100%	Ref	58%	Ref
Suburban	86%	0.3	84%	0.8	73%	0.9	100%	0.9	55%	0.8
Rural	92%	0.06	86%	0.5	77%	0.6	99%	0.6	55%	0.8
FNS Region										
Mid Atlantic	73%	Ref	73%	Ref	63%	Ref	100%	Ref	43%	Ref
Midwest	89%	0.08	81%	0.3	75%	0.3	99%	0.8	52%	0.6
Mountain Plains	94%	0.04	85%	0.2	87%	0.04	99%	0.7	64%	0.2
Northeast	85%	0.2	79%	0.5	75%	0.2	100%	0.9	48%	0.7

Southeast	96%	0.07	94%	0.06	82%	0.2	100%	0.9	74%	0.1
Southwest	89%	0.06	86%	0.08	83%	0.04	100%	0.9	68%	0.1
West	80%	0.5	93%	0.03	55%	0.5	100%	0.9	41%	0.9
Location										
Inside Cafeteria	99%	Ref	97%	Ref	96%	Ref	100%	Ref	87%	Ref
School Store	82%	0.006	68%	<0.0001	57%	<0.0001	100%	0.9	16%	<0.0001
Other Locations	77%	<0.0001	88%	0.003	70%	<0.0001	99%	0.8	65%	<0.0001
Oversees Vending Machines										
Food Service	96%	Ref	90%	Ref	83%	Ref	100%	Ref	67%	Ref
Other	77%	0.003	79%	0.07	65%	0.01	99%	0.7	45%	0.04
Sweet snacks (n=665)										
Urbanicity										
Urban	85%	Ref	85%	Ref	92%	Ref	69%	Ref	60%	Ref
Suburban	80%	0.5	85%	0.9	94%	0.8	56%	0.03	59%	0.9
Rural	89%	0.6	91%	0.3	96%	0.6	56%	0.07	60%	0.9
FNS Region										
Mid Atlantic	83%	Ref	72%	Ref	97%	Ref	62%	Ref	42%	Ref
Midwest	79%	0.7	84%	0.3	91%	0.5	48%	0.2	54%	0.4
Mountain Plains	99%	0.3	94%	0.08	96%	0.9	68%	0.6	70%	0.1
Northeast	91%	0.6	95%	0.08	93%	0.7	67%	0.7	68%	0.1
Southeast	97%	0.4	99%	0.03	99%	0.6	64%	0.9	80%	0.06
Southwest	99%	0.2	85%	0.2	99%	0.3	67%	0.7	67%	0.08
West	47%	0.01	76%	0.8	71%	0.03	48%	0.3	35%	0.7
Location										
Inside Cafeteria	90%	Ref	98%	Ref	86%	Ref	96%	Ref	84%	Ref
School Store	91%	0.9	84%	0.08	99%	0.0006	16%	<0.0001	32%	<.0001
Other Locations	74%	0.0001	79%	<0.0001	85%	0.7	69%	<0.0001	63%	<.0001
Oversees Vending Machines										
Food Service	88%	Ref	98%	Ref	95%	Ref	60%	Ref	70%	Ref
Other	82%	0.05	76%	0.007	93%	0.8	61%	0.9	49%	0.04

*Includes products with artificial sweeteners that are only compliant in high schools.

[1] Among n= 72 middle and high schools in a national sample of schools selling competitive foods.

[2] “Other” includes: (1) a grain product that contains 50 percent or more whole grains by weight (have a whole grain as the first ingredient); (2) a product with the first ingredient being a fruit, a vegetable, a dairy food, or a protein food; or (3) a combination food that contains at least ¼ cup of fruit and/or vegetable

[3] Milk includes unflavored or flavored fat-free milk, low-fat milk, and milk alternatives as permitted by the National School Lunch Program /School Breakfast program. Compliance for milk products is ≤ 12 fluid ounces.
[4] Compliance for 100% fruit juice is ≤ 12 fluid ounces.
[5] Low and no-calorie beverages (high school only) include low calorie drinks with the equivalent of 5 calories per fluid ounce (maximum of 60 kcals/12 fl oz) and no-calorie beverages with less than 5 calories per 8 fluid ounces (maximum of 10 calories/20 fl oz).
[6] Includes combinations of fruit or vegetables with dips or nut butters (e.g., apples with peanut butter)
